# Experimental Study on Atomization Characteristics of Gas–Liquid Two-Phase Flow Nozzle and Its Dust Removal Effect

**DOI:** 10.3390/ma15020565

**Published:** 2022-01-12

**Authors:** Xueming Fang, Bingyou Jiang, Liang Yuan, Yuxiang Liang, Bo Ren, Wenhan Tao, Xianbao Li

**Affiliations:** 1 Key Laboratory of Industrial Dust Control and Occupational Health, Ministry of Education, Anhui University of Science and Technology, Huainan 232001, China; 2016120755@jou.edu.cn (X.F.); Z20139286@stu.haut.edu.cn (W.T.); 2 National & Local Joint Engineering Research Center of Precision Coal Mining, Anhui University of Science and Technology, Huainan 232001, China; 3 State Key Laboratory of Mining Response and Disaster Prevention and Control in Deep Coal Mines, Anhui University of Science and Technology, Huainan 232001, China; 4 Guangdong Heer Environmental Technology Co., Ltd., Foshan 528000, China; 15030720551@xs.hnit.edu.cn; 5 School of Emergency Management and Safety Engineering, China University of Mining and Technology, Beijing 100083, China; 631605010426@mails.cqjtu.edu.cn; 6 Coal Mining National Engineering Technology Research Institute, Huainan 232001, China; 7 Pingxiang Anyuan Ventilation Equipment Co., Ltd., Pingxiang 337000, China; 16517210472@stumail.sdut.edu.cn

**Keywords:** gas–liquid two-phase flow nozzle, respirable dust, condensed capture, ambient humidity

## Abstract

An experimental study on the flow rate and atomization characteristics of a new gas–liquid two-phase flow nozzle was carried out to use high-concentration respirable dust in the workplace of high-efficiency sedimentation coal production based on the gas–liquid two-phase flow nozzle technology. The simulation roadway of dust fall in large coal mines was constructed, and the respirable rock dust produced by fully mechanized mining surfaces was chosen as the research object. The effects of humidity on the capture effect of respirable rock dust were analyzed in the experimental study. The results demonstrated that: (1) the distribution range of the particle size of fogdrops declines with the reduction in fogdrops D_50_, D_[3,2]_ and D_[4,3]_, which are produced by gas–liquid two-phase flow nozzles. (2) The initial ambient humidity in the simulated roadway was 64.8% RH. After the gas–liquid two-phase flow spray was started, the ambient humidity was elevated by 23.2 to 23.5% RH within 840s and tended to be stable and no longer grew after reaching 88.0–88.3% RH. The initial growth rate of the ambient humidity in the simulated roadway was high, and then was gradually slowed down. (3) Humidity is an important factor influencing the collection of respirable dust. The humidity at 10.0 m leeward of the dust-producing point was increased by 19.6% RH, and the sedimentation rate of respirable dust was increased by 6.73%; the two growth rates were 13.1% RH and 9.90% at 20.0 m; 16.4% RH and 15.42% at 30.0 m; 18.4% RH and 11.20% at 40.0 m. In practical applications of the gas–liquid two-phase flow nozzle in coal mining activities, attention shall be paid to not only the influences of its atomization characteristics on the capture effect of respirable dust but also the influences of the flow rate of the nozzle on the humidity of the working surface. Appropriate gas and water supply pressures shall be chosen according to the space and respirable dust concentration on the working surface to realize a better dust removal effect.

## 1. Introduction

Recently, dust pollution of the fully mechanized mining surface in underground coal mines has become increasingly prominent with the continuous improvement of the mechanization level and gradual growth of mining intensity [1,2,3,4]. These dusts, especially respirable dusts, threaten the physical health of workers and production safety in coal mining activities [5,6,7]. According to the 2020 revised edition of the Coal Mine Safety Regulations of the People’s Republic of China, underground operating locations, such as the conveyor transfer point, must be installed with a spray device or duct collector, and the spray dust sedimentation or dust removal by dust collector is needed. Gas–liquid two-phase flow spray is a new type of spray mode taking compressed air and pressured water as double powers [8,9,10]. In comparison to the conventional high-pressure spraying mode, this spray mode has various advantages, such as a good atomization effect, low requirements for atmospheric pressure and hydraulic pressure and small water consumption. The gas–liquid two-phase flow nozzle has a good effect in the capture of dust, especially to respirable dust [10,11,12,13,14].

The current research on the mechanism of gas–liquid two-phase flow spraying dust reduction in coal fields mainly focuses on inertial collision dust trapping and diffused trapping. Inertial collision is generally used to trap dust with a particle size greater than 0.5 µm. Under the effect of inertia, dust particles and water drop particles stick together after mutual collision and settle rapidly due to gravity, thereby achieving dust reduction. It is generally accepted by scholars that the relative velocity of respirable dust particles to liquid droplets, density and diameter of dust particles and diameter of liquid droplets are the primary factors influencing the dust collection efficiency of gas–liquid two-phase flow spray [15,16,17,18]. The effect of inertial collision is weakened for dust particles with a small mass and a diameter of less than 0.5 µm. Therefore, diffused trapping becomes an important method of dust trapping. Related studies have been carried out on this basis, and it has been found that the ambient humidity in the roadway is the main factor influencing the respirable dust collection efficiency of gas–liquid two-phase flow spray. When the water content in the air is nearly saturated, the dust particles in the air will become condensation nuclei, and a layer of liquid film will be formed on the surface. The dust particles have been moistened by water before contact with water drops; hence, the diameter of the particles is enlarged, and the dust trapping effect is improved. In order to quantitatively study the effect of ambient humidity on the dust sedimentation of gas–liquid two-phase flow spray, the analysis and discussion were implemented on an experimental basis, expecting to guide the practical application of gas–liquid two-phase flow spray in the field of dust sedimentation of coal mines.

## 2. Design of Experimental System

### 2.1. Nozzle Atomization Performance Test System

**Figure 1:**
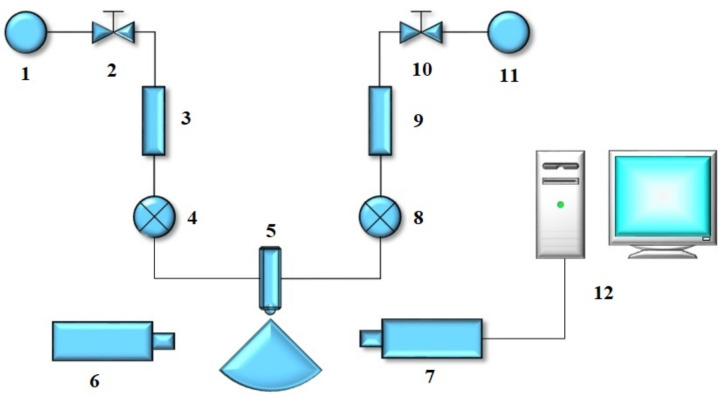
Nozzle atomization performance test system. 1—water source; 2—water pressure stabilization control valve; 3—liquid glass rotameter; 4—liquid pressure gauge; 5—experimental nozzle; 6—laser transmitter of laser particle sizer; 7—laser receiver of laser particle sizer; 8—gas pressure gauge; 9—gas glass rotameter; 10—gas pressure reducing and stabilizing valve; 11—gas cylinder; 12—data processing computer.

For the purpose of testing the atomization characteristics of the new gas–liquid two-phase flow atomization nozzle, running water was used as the liquid phase medium, and the air was used as the gas phase medium. The schematic diagram of the experimental system is shown in Figure 1. It is mainly composed of a water supply device, air supply device, test device, injection device and other parts.

The water supply system and the air supply system, respectively, provided clean and pressurized water and compressed air to the injection system during the experiment. The water and compressed air entered the nozzle from the water inlet end and the air inlet end, respectively, and were mixed in the mixing chamber of the nozzle. Under the effect of compressed air, the water was injected from the spraying nozzle through atomization. After the atomization was stable, the atomization characteristics of the atomization area were tested and recorded with the test system. Besides, the data, including the pressure, atomization volume of steam, and air consumption, were recorded.

### 2.2. Large Dust Reduction Experimental Tunnel System

**Figure 2:**
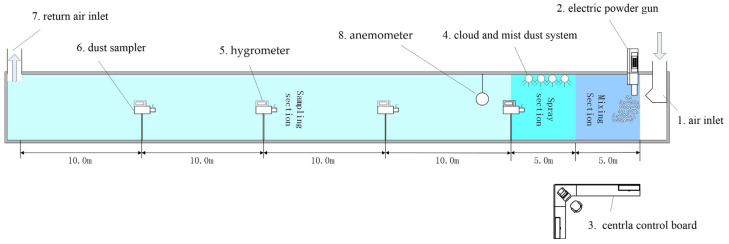
Dust removal experimental system of gas–liquid two-phase flow nozzle.

**Figure 3:**
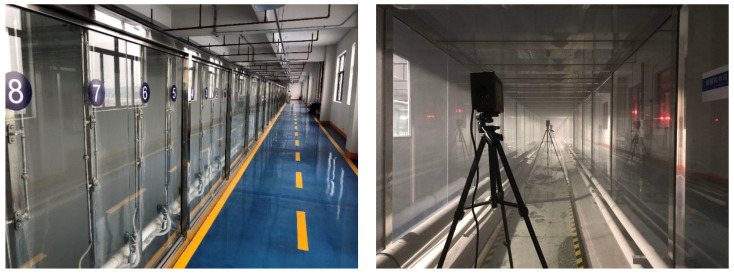
Dust removal of simulation roadways of gas–liquid two-phase flow nozzle.

By reading the related literature, it has been found that the area of the roadway section involved in most laboratory simulations of dust sedimentation is 1.8–2.5% of the actual roadway sectional area. The simulation test of dust sedimentation in a small-scale simulated roadway has natural deficiencies; for example, the flow field distribution in a small-scale simulated roadway is greatly different from that in an actual excavation roadway, and the natural sedimentation rates of dust, especially respirable dust, in the two roadways are also different. Besides, the spray droplets present different diffusion trends in the two [19,20]. An experimental system of gas–liquid two-phase spray dust sedimentation, which could simulate the ventilation, dust production, etc., in the excavation roadway of coal mine, was designed by adhering to the concepts of improving the experimental safety and stability and enhancing the scientificity of the experimental data and the principle of high compliance with the actual excavation roadway (Figure 2 and Figure 3).

This dust sedimentation experimental system was mainly composed of an air supply/exhaust module, dust-producing module, gas–liquid two-phase flow spray module, data acquisition module and central control console. The air supply/exhaust module included a permanent magnetic variable-frequency screw air compressor, centrifugal fan, etc. The dust-producing module contained an electric powder sprayer, respirable dust producer, etc. The gas–liquid two-phase flow spray module included a storage water tank, high pressure water pump, gas–liquid two-phase flow nozzle, pipeline, pressure gauge, etc. The data acquisition module included a dust sampler, anemometer, thermometer, hygrometer, data acquisition device, etc. All the above modules were connected to the central control console. The experimenter could control the start/stop of all the devices and regulate the wind velocity in the simulated roadway and gas and water supply pressures via the central control console. The cross-sectional area and length of the main part of the simulated roadway were 2.0 × 2.0 m and 50.0 m, respectively, and both the air inlet and air outlet were located at the two ends of the main body of the simulated roadway. The floor of the simulated roadway was paved using 304 stainless steel, a reflux tank was set to prevent the sewage accumulation and double-layer armored glass was used at both sides of the simulated roadway, which made it convenient for the experimenter to observe the migration status of the dust and spray in the roadway while ensuring safety. The dust producer was located at the air inlet of the simulated roadway, and the gas–liquid two-phase flow nozzle was the new-type low-energy-consumption and high-throughput nozzle co-designed and manufactured by Key Laboratory of Shock Wave Physics and Detonation Physics of China Academy of Engineering Physics and Xiangtan University.

## 3. Experimental Schemes

Experiments were designed to explore the influence of humidity on the dust sedimentation laws of gas–liquid two-phase flow spray, as seen in Table 1.

The experimental group 1 is an experiment on flow characteristics of the gas–liquid two-phase flow nozzle to discuss the relationship between flow rate and water supply pressure. The water consumptions and gas consumptions of the nozzle under 6 gas supply pressures (0.15 MPa, 0.18 MPa, 0.20 MPa, 0.25 MPa, 0.27 MPa and 0.29 MPa) when the water supply pressure is 0.2 MPa were tested by an electromagnetic flowmeter and air mass flowmeter.

The experimental group 2 is an experiment on atomization characteristics of gas–liquid two-phase flow nozzle, which involved atomization parameters of D_25_, D_50_, D_75_, D_90_, D_[3,2]_ and D_[4,3]_. The water supply pressure of the nozzle was kept constant at 0.20MPa and the gas supply pressure was adjusted to 0.15 MPa, 0.18 MPa, 0.20 MPa, 0.25 MPa, 0.27 MPa and 0.29 MPa. The section center at 30.0 cm in front of the mouth of gas–liquid two-phase flow nozzle was chosen as the sampling point of fogdrop performance parameters. The atomization characteristics of the nozzle under different working conditions were tested by the OMEC DP-02 spray size analyzer.

The experimental group 3 discusses influences of gas–liquid two-phase flow nozzle on variations of air humidity in the simulation roadway. Hygrometers were set at 10.0 m, 20.0 m, 30.0 m and 40.0 m away from the dust source in the simulation roadway. The height between the hygrometers and the floor of the simulation roadway was 1.5m and the distance to the side wall was 1.0 m. Turn on the gas–liquid two-phase flow nozzle system and the water pressure was set at 0.20 MPa and the gas pressure was set 0.29 MPa. The wind speed in the roadway was adjusted to 0.5 m/s and changes in air humidity at different positions in the roadway were measured.

The experimental group 4 tests dust removal effect of gas–liquid two-phase flow nozzle under different humidity values. The dust removal effect of the gas–liquid two-phase flow nozzle in different periods when the gas and water supply pressures are 0.29 Mpa and 0.20 Mpa was measured. In the experiment, inert rock powder with D_50_ = 1.57 μm (major ingredient is SiO_2_) was used as the sample. The independent-made electronic powder gun was used to spray dusts. The initial concentration of dust in air in the roadway was kept at 44.67 mg/m^3^ by adjusting the input gas pressure of the electronic powder gun. A total of four dust concentration samplers were installed at 10.0 m, 20.0 m, 30.0 m and 40.0 m from the dust source in the simulation roadway to collect respirable dust samples in the roadway. The sampling time was 1min and the sampling flow rate was 20 L/min. Variable frequency regulation was applied to the fan and wind speed in the measurement section of the simulation roadway was stabilized at 0.5 m/s.

## 4. Experimental Results and Analysis

### 4.1. Flow Rate Analysis of the Gas–Liquid Two-Phase Flow Nozzle

**Figure 4:**
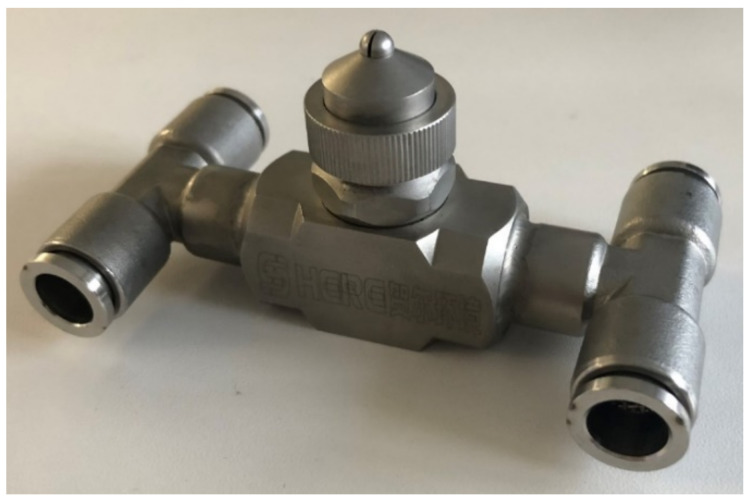
Gas–liquid two-phase atomizing nozzle.

The gas–liquid two-phase atomizing nozzle was used in the experiment (Figure 4). Water flows that entered the nozzle were atomized into small-sized water mist in a solid cone. The size of the atomized particle and flow rate of the nozzle were controlled by regulating the gas pressure and water pressure.

**Figure 5:**
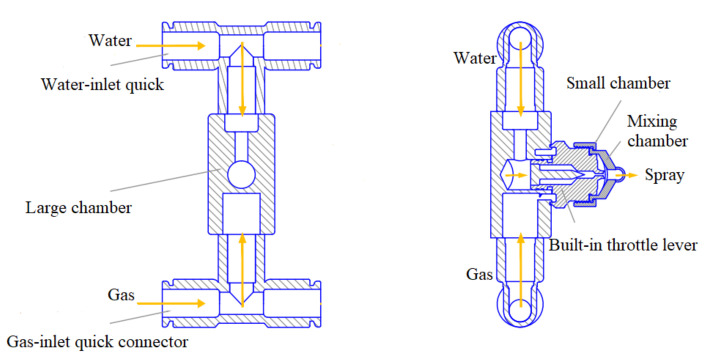
The working principle of the gas–liquid two-phase atomization nozzle.

The internal structure of the nozzle is shown in Figure 5. The working principle of the gas–liquid two-phase atomization nozzle is that the water and air entered the atomization nozzle from the water-inlet quick connector and gas-inlet quick connector. Water entered the large chamber through the flow channel, and the large channel was provided with a throttle lever to limit the flow and control the speed of the water, increase the relative speed between the water and gas, maximize the atomization effect of the gas to the water in unit area, and achieve a good atomization effect of the two-phase flow nozzle under low air pressure. At the same time, air entered the small chamber from the flow channel. In the small chamber, the air hole was provided with a deviation angle. There was a certain angular deflection in axial and horizontal directions to form a rotational flow channel. Then, the eddy airflow effect was produced to increase the flow rate of gas. The airflow moved clockwise at a high speed along the gas flow channel to enter the mixing chamber and directly impact liquid from the water hole in the gas–liquid mixing chamber. With mutual impact, squeezing, shearing, and other effects between the gas and the liquid, water was crushed to slight fogdrops. In addition, the mixing chamber was equal to a resonance chamber. High-speed gas functioned in the mixing chamber back and forth to produce ultrasonic resonance. With the pulling effect of ultrasonic waves, water was further refined to complete initial atomization. At last, the convergence of gas–liquid two-phase flow was accelerated through the swelling site of the fog outlet. The liquid drops were refined after further absorbing the kinetic energy of the gas. Therefore, secondary atomization was formed at the fog outlet.

**Figure 6:**
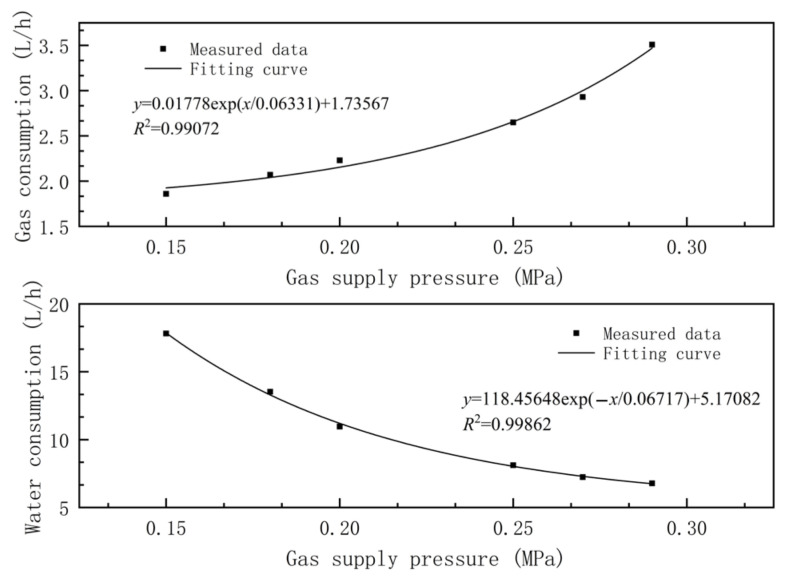
Effects of gas supply pressure on gas consumption and water consumption of the nozzle.

When the water supply pressure of the nozzle is kept constant and the gas supply pressure increases, the gas consumption of the nozzle increases continuously with the increase in the gas supply pressure. Meanwhile, the gas–liquid two-phase flows in the atomizing chamber increase as the gas supply pressure increases and resistance at the end of the water inlet increases, while the water consumption of the nozzle decreases. The flow rate of the gas–liquid two-phase flow nozzle when the water supply pressure was fixed at 0.20 MPa and the gas supply pressure was different was tested. The relation curve between flow rate of the nozzle and gas supply pressure is shown in Figure 6. Clearly, when the water supply pressure was kept at 0.20 MPa, the gas consumption of the gas–liquid two-phase flow nozzle increased as the gas supply pressure increased and the water consumption decreased continuously.

For this gasliquid two-phase atomizing nozzle, the relation fitting equation between flow rate and gas supply pressure when the water supply pressure is 0.20 MPa is:

(1) $$Q_{air}=0.01778\exp(p_{air}/0.06331)+1.73567$$

(2) $$Q_{L}=118.45648\exp(-p_{air}/0.06717)+5.17082$$

where $Q_{air}$ is the gas consumption, $Q_{L}$ is the water consumption, and $p_{air}$ is the air supply pressure.

The fitting curve correlation coefficients of flow rate of the nozzle were all higher than 0.99, proving the high correlation between the flow rate and gas supply pressure. It can be seen from the fitting curve that, when the water supply pressure is 0.20 MPa, the gas consumption increases exponentially as the gas supply pressure increases. According to the fitting curve between water consumption and gas supply pressure, the water consumption of the gas–liquid two-phase flow nozzle attenuates exponentially with the increase in gas supply pressure when the water supply pressure is 0.20 MPa.

### 4.2. Atomization Characteristics of the Gas–Liquid Two-Phase Flow Nozzle

**Figure 7:**
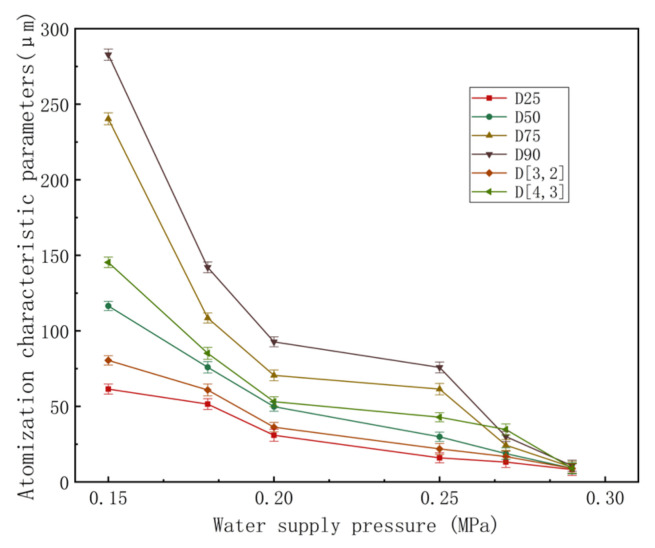
Atomization characteristic parameters of the gas–liquid two-phase flow nozzle.

The size distributions of the mist particles that are produced by the gas–liquid two-phase flow nozzle when the water supply pressure is 0.20 MPa and the gas supply pressure is 0.15 MPa, 0.18 MPa, 0.20 MPa, 0.25 MPa, 0.27 MPa and 0.29 MPa are shown in Figure 7. Obviously, all the parameters that describe the mist particle size, including D_25_, D_50_, D_75_, D_90__,_ D_[3,2]_ and D_[4,3]_, decrease with the increase in the gas supply pressure from 0.15 MPa to 0.29 MP when the water supply pressure is 0.20 MPa.

**Figure 8:**
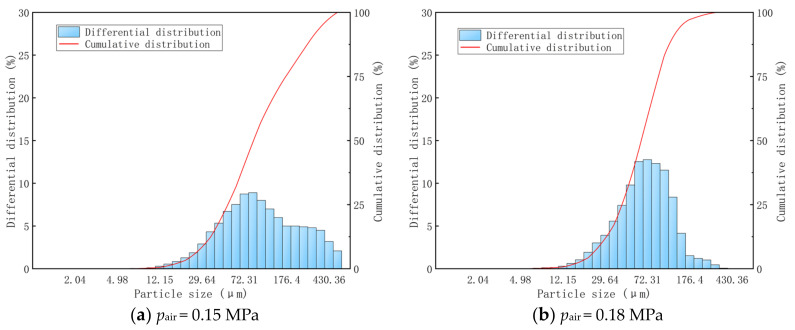

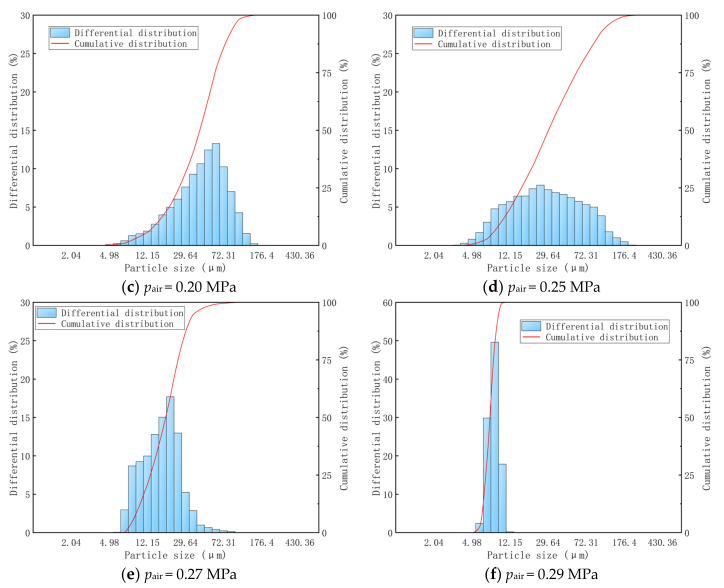
Size distribution of fogdrop particles under different gas supply pressures.

Figure 8a–f shows the integral distribution and cumulative distribution of the particle size of the fogdrop through water atomization by the gas–liquid two-phase flow nozzle under different air pressure conditions (0.15 Mpa, 0.18 MPa, 0.20 MPa, 0.25 MPa, 0.27 MPa, 0.29 MPa). The differential distribution histogram of fogdrop particles in Figure 8 shows that, when the water supply pressure remained constant at 0.20 MPa with the increase in the air supply pressure, the peak for the differential distribution of the fogdrop particles gradually moved to the right, that is, the direction of reduced fogdrop particle size. When the air pressure was between 0.15 MPa and 0.25 MPa, the distribution range of the fogdrop particle size was wide. When the air pressure was 0.27 MPa, the particle size of the fogdrop produced by the nozzle was distributed between 7.11 μm and 86.43 μm. When the air pressure was 0.29 MPa, the fogdrop particle size distribution range was only between 5.95 μm and 14.52 μm.

### 4.3. Variations of Humidity in the Simulation Roadway

**Figure 9:**
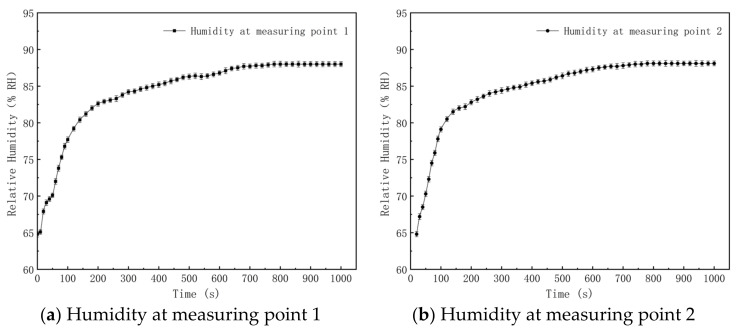

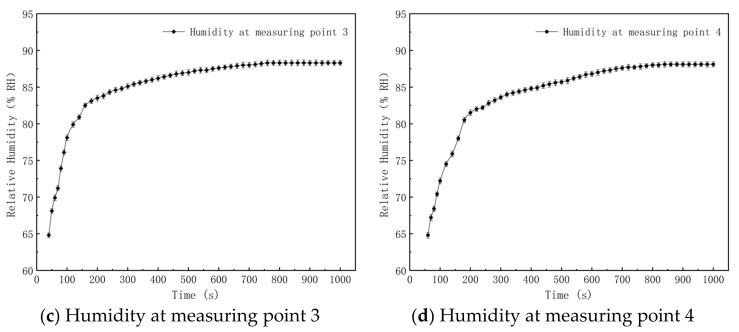
Variations in humidity at different measuring points in the simulation roadway.

An experiment on the effects of the gas–liquid two-phase flow nozzle on the humidity in the simulation roadway was carried out. The water pressure and gas pressure of the gas–liquid two-phase flow nozzle were set at 0.20 MPa and 0.29 MPa, and the wind speed of the roadway was regulated as 0.5 m/s. Variations in the air humidity at four measuring points, which are 10.0 m (point 1), 20.0 m (point 2), 30.0 m (point 3) and 40.0 m (point 4) away from the dust source in the simulation roadway, are shown in Figure 9.

It can be seen from Figure 9 that the overall variation trends of humidity at four measuring points in the simulation roadway are the same, and the air humidity at four measuring points increases as time goes on. Under the same initial humidity of 64.8% RH at different points, the ambient humidity values at the measuring points one, two, three and four were increased by 18.5% RH, 18.8% RH, 19.5% RH and 18.0% RH, respectively, at 260 s in comparison with the initial humidity, and by 23.2% RH, 23.3% RH, 23.5% RH and 23.3% RH, respectively, at 840 s. This is because the gas–liquid two-phase flow nozzle system in the simulation roadway has been in the working state all the time. Fogdrop particles evaporate continuously after they enter into the roadway through the nozzle, so the air humidity of the roadway increases continuously. The growth rate of the air humidity at the measuring points is relatively high in the beginning, but it decreases gradually and finally tends to be stable. With the continuous evaporation of fogdrops, the temperature in the roadway decreases gradually, and the difference between the practical vapor pressure in the roadway and saturated vapor pressure declines gradually. According to the Dalton law, the evaporation rate of fogdrops decreases. In other words, the growth rate of the air humidity decreases gradually and the air humidity in the roadway is constant. Air humidity values at four measuring points begin to change at different times. After the nozzle system is turned on, air and fogdrop particles with a high humidity enter into the simulation roadway through the nozzle. The air humidity at measuring point one increases accordingly. The air humidity at measuring points two, three and four begins to increase at 20 s, 40 s and 60 s after the turning on of the nozzle system. This is because measuring points two, three and four are at the downwind side of measuring point one and they are 10.0 m, 20.0 m and 30.0 m away from measuring point one. The airflow rate in the simulated tunnel is 0.5 m/s. Hence, the time for the arrival of air that has a high content of fogdrops at adjacent measuring points is 20 s.

### 4.4. Respirable Dust Capture Effect of the Gas–Liquid Two-Phase Flow Nozzle

Previous studies have quantitatively analyzed the factors affecting the efficiency of inertial collision. The relationship between the coefficient of inertia and the collision characteristics is defined as follows.

(3) $$\varphi =\frac{K\rho_{P}vd_{P}}{18\mu D}$$

In Equation (3), *K* is the collision probability coefficient, $\rho_{P}$ is the density of the dust particles, *v* is the relative velocity of the dust particles and water droplets, $d_{P}$ is the diameter of the dust particles, *μ* is the aerodynamic viscosity coefficient and *D* is the diameter of the water droplets.

The larger the inertia coefficient, the higher the collision probability of the dust and water droplets. Equation (3) shows that the coefficient of inertia is proportional to the relative velocity and diameter of the dust particles and is inversely proportional to the diameter of the droplets.

In the simulation roadway, respirable dust in the air was sampled by the dust sampler, thus enabling to calculate the respirable dust capture efficiency of the gas–liquid two-phase flow nozzle. We switched on the electric dusting machine and the fan to keep the total mass of respirable dust entering the tunnel, the angle and speed of dust injection and the wind speed in the tunnel within the unit time unchanged. After 15 min, the concentration of airborne respirable dust in the tunnel stopped increasing and was stable at 44.67 mg/m^3^, which was recorded as *C*_0_.

After enabling the atomization function, the calculation formula for the average concentration *C*_1_ of respirable dust at the sampling point is as follows:

(4) $$C_{1}=\frac{1000\left(m_{1}-m_{0}\right)}{Qt}$$

where

*m*_0_—mass of filter membrane before sampling (mg);
*m*_1_—mass of filter membrane after sampling (mg);
Q—sampling flow rate (L/min);
*t*—sampling time, (min).

The sedimentation rate of respirable dust at the sampling point is recorded as *η*, then

(5) $$\eta =\frac{C_{0}-C_{1}}{C_{0}}\times 100\%$$

**Figure 10:**
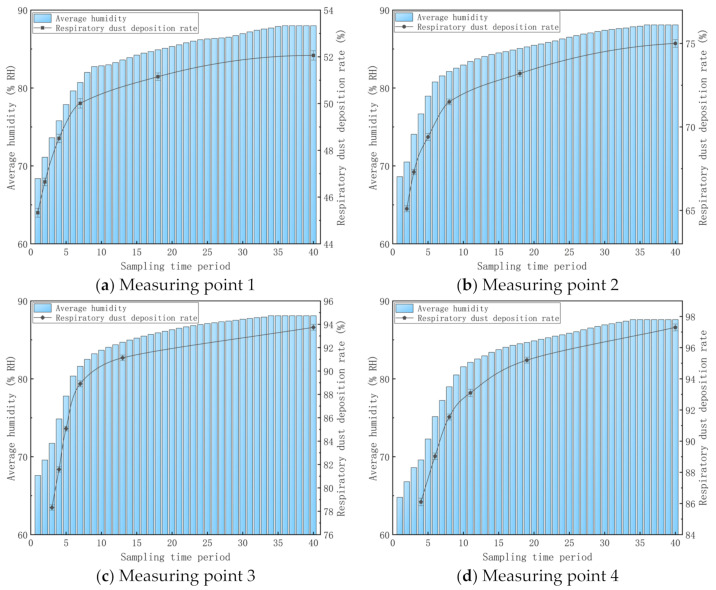
Variations in air humidity and respirable dust sedimentation rate in the simulation roadway.

In the dust sampling process, the humidity in the simulation roadway changes continuously. Hence, the sampling period of the dust sampler (60 s) was chosen as the length of an interval, and the mean humidity at different measuring points in 60 s was calculated. The interval between every two intervals is 20 s. The 0–60 s range was recorded as interval one, and 20-80 s was recorded as interval two. The rest can be done in the same way. Hence, the air humidity variations at different measuring points can be shown in Figure 10.

It can be seen from Figure 10 that the mean humidity of the four measuring points increases continuously as time goes on. The growth rate of the mean humidity is relatively high in the beginning, but it decreases gradually. The mean humidity values at measuring point one in six intervals of 0–60 s, 20–80 s, 60–120 s, 120–180 s, 340–400 s and 780–840 s are 68.4% RH, 71.1% RH, 75.8% RH, 80.7% RH, 84.9% RH and 88.0% RH, respectively. These six intervals were chosen as the respirable dust sampling time at measuring point one. The mean humidity values at measuring point two in six intervals of 20–80 s, 40–120 s, 80–140 s, 140–200 s, 340–400 s and 780–840 s are 70.5% RH, 74.1% RH, 78.9% RH, 82.1% RH, 85.0% RH and 88.1% RH, respectively. These six intervals were used as the sampling time of respirable dust at measuring point two. The mean humidity values at measuring point three in six intervals of 40–100 s, 60–120 s, 80–140 s, 120–180 s, 240–300 s and 780–840 s are 71.7% RH, 74.9% RH, 77.8% RH, 81.6% RH, 84.7% RH and 88.3% RH, respectively. These six intervals were used as the sampling time of respirable dust at measuring point three. The mean humidity values at measuring point three in six intervals of 60–120 s, 100–160 s, 140–200 s, 200–260 s, 360–420 s and 780–840 s are 69.6% RH, 75.1% RH, 79.0% RH, 82.1% RH, 84.7% RH and 88.0% RH, respectively. These six intervals were used as the sampling time of respirable dust at measuring point four.

The fan and electronic powder gun in the simulation roadway are turned on. After the airflow in the roadway becomes stable, the gas–liquid two-phase flow nozzle is turned on and begins to timing. Six samples were collected at four measuring points, respectively. The sampling times were set as mentioned above. After repeated multiple samplings, the sedimentation rates of respirable dust at different measuring points are shown in dotted lines in Figure 10. It can be seen from curve variations in Figure 10 that:

(1) When the respirable dust concentrations at dust production points in the roadway are the same and the wind speeds are the same, the gas pressure and water pressure of the gas–liquid two-phase flow nozzle system were kept constant. As the air humidity in the simulation roadway changes, the respirable dust concentration in the air at the same measuring point may change. In other words, the respirable dust capture efficiency of gas–liquid two-phase flow nozzle changes. It is proved that air humidity influences the respirable dust capture efficiency of the gas–liquid two-phase flow nozzle significantly when it is used to produce water mist (D_50_ = 9.00 μm) in a roadway. The sedimentation rates of respirable dust at four measuring points are positively related with air humidity. When the air humidity in the simulation roadway increases, the respirable dust concentration decreases and the sedimentation rate of respirable dust increases.
(2) When the mean air humidity at measuring point one is 68.4% RH, the sedimentation rate of respirable dust in this time interval is 45.33%. When the mean air humidity is 88.0% RH, the sedimentation rate of respirable dust in this time interval is 52.06%. From the beginning working of the gas–liquid two-phase flow system to the simulation roadway, air humidity tends to be stable. The air humidity at measuring point one is increased by 19.6% RH and the sedimentation rate of respirable dust is increased by 6.73%. At measuring point one, humidity variations influence the dust capture efficiency slightly. At measuring point one, humidity changes influence the dust capture efficiency slightly. This is because the relative kinematic velocity between the respirable dust particles and fogdrop particles in the air is relatively large and collision capture is the main reason for dust sedimentation. The sedimentation of dust is mainly achieved by mutual collisions among fogdrop particles, dust-containing fogdrop particles and respirable dust, thus forming large particles. Hence, dust sediments on the bottom of the simulation roadway or adheres onto the inner wall of the simulation roadway.

When the mean air humidity at measuring point two is 70.5% RH, the sedimentation rate of respirable dust in this time interval is 65.10%. When the mean air humidity is 88.1% RH, the sedimentation rate of respirable dust in this time interval is 75.00%. The air humidity at measuring point two is increased by 13.1% RH and the sedimentation rate of respirable dust is increased 9.90%. At measuring point two, the influences of humidity changes on respirable dust capture efficiency are relieved to some extent. This is because vapor in air uses dust particles as the cohesion nucleus and dust-containing fogdrop particles are formed with a diameter far higher than the size of respirable dust particles. Hence, the possibility of forming greater particles through the collision between these respirable dust particles and fogdrop particles is increased and the effects of condensed capture on respirable dust sedimentation are improved to some extent.

When the mean air humidity at measuring point three is 71.7% RH, the sedimentation rate of respirable dust in this time interval is 78.32%. When the mean air humidity is 88.1% RH, the sedimentation rate of respirable dust in this time interval is 93.74%. The air humidity at measuring point three is increased by 16.4% RH and the sedimentation rate of respirable dust increases by 15.42%. At measuring point three, the influences of humidity changes on respirable dust capture efficiency are intensified significantly. Since the relative speed between dust particles and fogdrop particles is small, the probability for direct collision among fogdrop particles, dust-containing fogdrop particles and respirable dust decreases. The collision capture and cohesion capture become the major capture modes of respirable dust.

When the mean air humidity at measuring point four is 69.6% RH, the sedimentation rate of respirable dust in this time interval is 86.10%. When the mean air humidity is 88.0% RH, the sedimentation rate of respirable dust in this time interval is 97.30%. The air humidity at measuring point four is increased by 18.4% RH and the sedimentation rate of respirable dust increases by 11.20%. At measuring point four, the influences of humidity changes on respirable dust capture efficiency are relieved to some extent. Since the respirable dust concentration in the air in the simulation roadway is very low, the respirable dust concentration in the air of the simulation roadway is low according to characteristics of dust capture, thus making the dust more difficult to sediment under the effect of water mist.

## 5. Conclusions

(1) For the new gas–liquid two-phase flow nozzle in the experiment, the gas consumption of the nozzle increases exponentially when the gas supply pressure increases at the water supply pressure of 0.20 MPa. Moreover, the water consumption of the nozzle decreases exponentially with the increase in the gas supply pressure.
(2) The water supply pressure of the gas–liquid two-phase flow nozzle is kept constant. D_25_, D_50_, D_75_ and D_90_ all decrease gradually with the increase in the gas supply pressure. When the gas pressure is 0.15–0.25 MPa, the size distribution range of the fogdrop particles is wide. When the gas pressure is 0.27 MPa, the size distribution of the fogdrop particles that are produced by the nozzle is 7.11–86.43 μm. When the gas pressure is 0.29 MPa, the size distribution range is only 5.95–14.52 μm.
(3) After the gas–liquid two-phase flow spray was started in the simulated roadway, the humidity values at four measuring points (10.0 m, 20.0 m, 30.0 m and 40.0 m) away from the dust source were increased with time. Under the same initial humidity of 64.8% RH, the ambient humidity values at the measuring points one, two, three and four were increased by 18.5% RH, 18.8% RH, 19.5% RH and 18.0% RH, respectively, at 260 s in comparison with the initial humidity, and by 23.2% RH, 23.3% RH, 23.5% RH and 23.3% RH, respectively, at 840s. The growth rate of ambient humidity was fast within the first 260 s. Afterwards, it gradually slowed down and finally tended to be stable.
(4) It verifies the important role of humidity in respirable dust capture in the dust removal process of the gas–liquid two-phase flow nozzle. The higher the humidity is, the better the respirable dust capture efficiency of the nozzle will be. When the gas–liquid two-phase flow nozzle produces water mist (D_50_ = 9.00 μm), the humidity at the dust production point that is 10.0 m away from the downwind side is increased by 19.6% RH and the sedimentation rate of respirable dust increases by 6.73%. At 20.0 m away from the dust production point, the humidity is increased by 13.1% RH and the sedimentation rate of respirable dust increases by 9.90%. At 30.0 m away from the dust production point, the humidity is increased by 16.4% RH and the sedimentation rate of respirable dust increases by 15.42%. At 40.0 m away from the dust production point, the humidity is increased by 18.4% RH and the sedimentation rate of respirable dust increases by 11.20%.

In coal production activities, the gas–liquid two-phase flow nozzle was used to capture respirable dust. However, consideration has to be given not only to the effects of the spraying particle size on the capture of respirable dust but also the influences of the spraying quantity on the air humidity of the roadway. The dust removal effect can be improved by choosing appropriate gas and water pressure according to the cross-section size in practical roadways.

## Figures and Tables

**Table 1:** Experimental scheme.

Experiment No.	Experimental Content	Experimental Parameter
1	To determine the water consumption of gas–liquid two-phase flow nozzle	To study the influences of gas and water supply pressures on the water consumption of gas–liquid two-phase flow nozzle, and provide data support for experiment 3
2	To determine the atomization performance of gas–liquid two-phase flow spray, including D_50_, D_[3,2]_, D_[4,3]_, etc.	To determine the gas and water supply pressures used in the dust sedimentation experiment and provide data support for experiment 4
3	To determine the change of ambient humidity in the simulated roadway	To explore the influence of gas–liquid two-phase flow spray on the ambient humidity in the roadway and provide data support for experiment 4
4	To determine the respirable dust concentration in the simulated roadway	To simulate the influence of change in ambient humidity in the simulated roadway on the dust sedimentation of gas–liquid two-phase flow spray

## Data Availability

The data presented in this study are available on request from the corresponding author.
